# Supplementary material for: Self-organizing actin networks drive sequential endocytic protein recruitment and vesicle release on synthetic lipid bilayers
Source: bioRxiv. 2023 Feb 14:2023.02.14.528546. Preprint. [Version 1] doi: 10.1101/2023.02.14.528546 (PMC9949000; doi:10.1101/2023.02.14.528546)
Supplement: Supplement 9 [file NIHPP2023.02.14.528546v1-supplement-9.pdf]

bioRxiv preprint doi: https://doi.org/10.1101/2023.02.14.528546; this version posted February 14, 2023. The copyright holder for this preprint (which was not certified by peer review) is the author/funder, who has granted bioRxiv a license to display the preprint in perpetuity. It is made available under aCC-BY 4.0 International license.

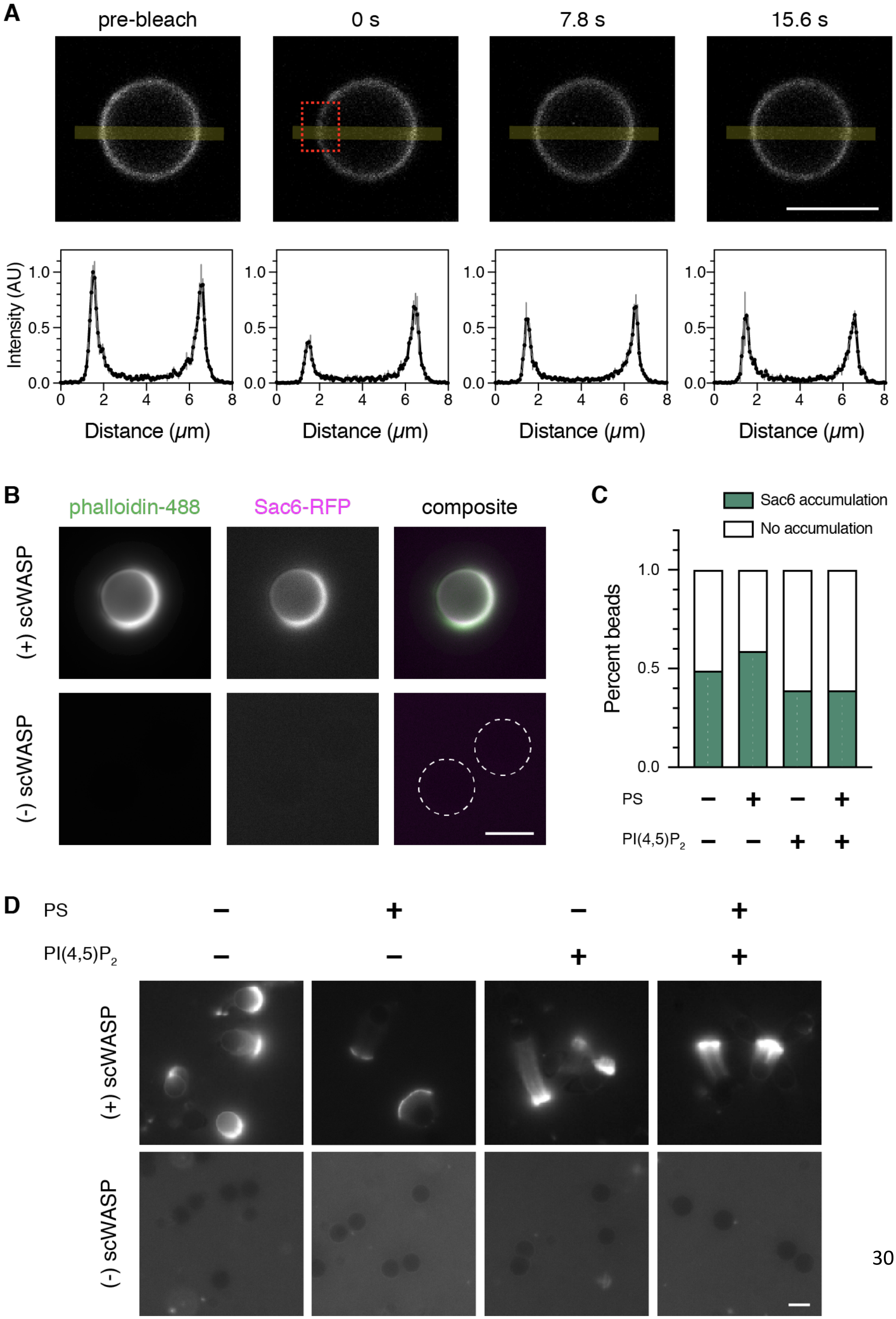

bioRxiv preprint doi: https://doi.org/10.1101/2023.02.14.528546; this version posted February 14, 2023. The copyright holder for this preprint (which was not certified by peer review) is the author/funder, who has granted bioRxiv a license to display the preprint in perpetuity. It is made available under aCC-BY 4.0 International license.

Figure S1. (A) Fluorescence recovery after photobleaching experiment of supported lipid bilayers containing 0.05% TexasRed-DHPE. Time-lapse confocal images and the corresponding fluorescence intensity of a line (yellow line) drawn through the bleaching region (red box) demonstrate rapid lipid turnover. (B) Phalloidin-488 (26.4µM) was added to scWASP-coated or uncoated bilayers (75% PC, 20% PS, 5% DGS-NTA) during incubation with cytoplasmic extract containing Sac6-RFP. Dotted lines outline the location of the beads lacking scWASP. (C and D) Sac6-GFP from yeast cytoplasmic extract labels actin networks assembled on scWASP-coated supported bilayers. Bilayer composition included PS and/or PI(4,5)P_2 as indicated. (C) Quantification of the percentage of beads with associated Sac6 fluorescence. At least 160 beads per sample were quantified. (D) Representative images of Sac6-GFP accumulation on bilayers. Scale bars, 5 µm.

bioRxiv preprint doi: https://doi.org/10.1101/2023.02.14.528546; this version posted February 14, 2023. The copyright holder for this preprint (which was not certified by peer review) is the author/funder, who has granted bioRxiv a license to display the preprint in perpetuity. It is made available under aCC-BY 4.0 International license.

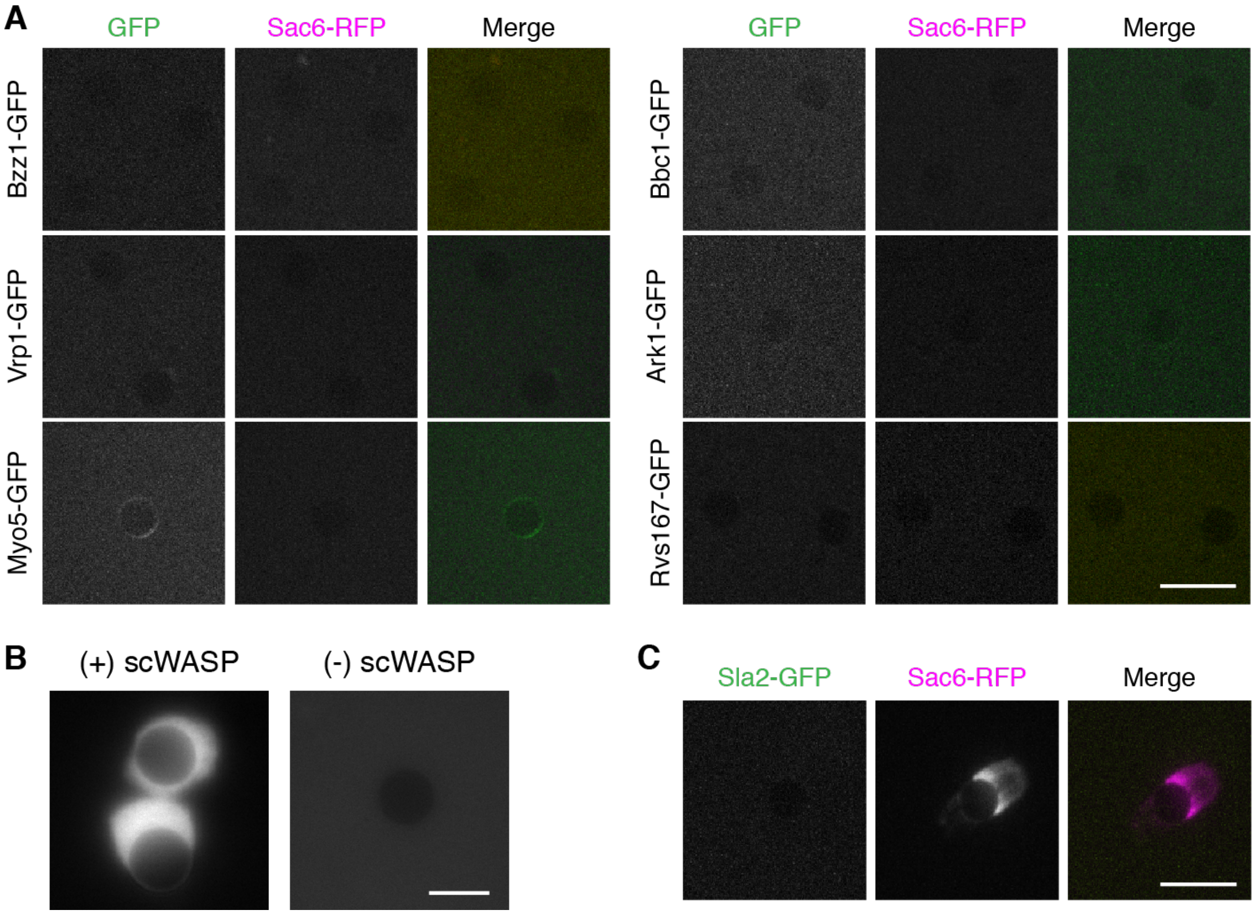

Figure S2. (A) Endocytic proteins do not localize to bilayers in the absence of scWASP. Extracts were prepared from strains expressing Sac6-RFP and the indicated endocytic proteins tagged with GFP, as in Figure 2B. Scale bar, 10 μm. (B) Abp140-GFP is recruited to actin networks. scWASP-coated and uncoated bilayers were incubated in cytoplasmic extract generated from a strain expressing Abp140-GFP. Scale bar, 5 μm. (C) Coat protein Sla2 does not localize to scWASP-coated bilayers. Bilayers were incubated in cytoplasmic extract generated from a strain expressing Sla2-GFP and Sac6-RFP. Scale bar, 10 μm. Representative fluorescence images from 3 independent experiments are shown.

bioRxiv preprint doi: https://doi.org/10.1101/2023.02.14.528546; this version posted February 14, 2023. The copyright holder for this preprint (which was not certified by peer review) is the author/funder, who has granted bioRxiv a license to display the preprint in perpetuity. It is made available under aCC-BY 4.0 International license.

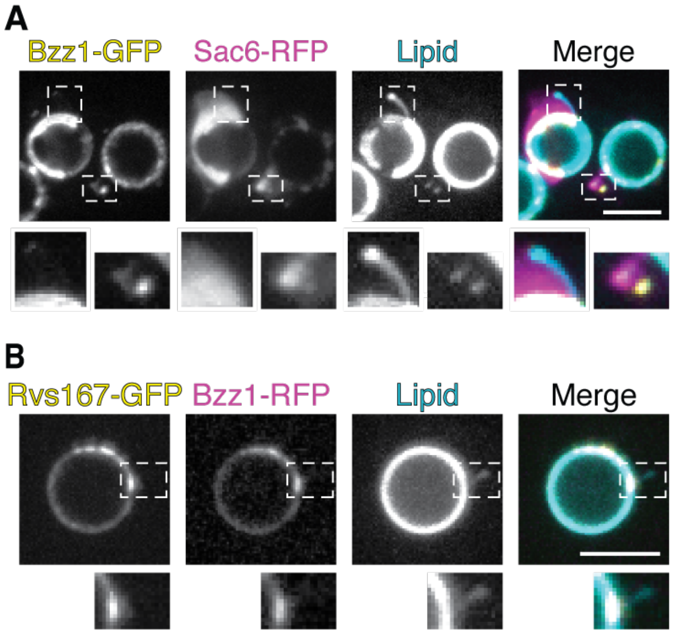

Figure S3. (A and B) scWASP-coated bilayers were incubated in cytoplasmic extract from strains expressing the indicated GFP- and RFP-tagged proteins. Maximum intensity projections of confocal stacks through the midsection of scWASP-coated bilayers containing lipid dye (0.5% MarinaBlue-DHPE) are displayed. Scale bars, 5 μm. (A) Insets show regions of an actin tail where membrane tubulation and membrane deformation occurred (lipid channel). (B) Inset shows membrane tubulation (lipid channel) at plaques of Rvs167-GFP and Bzz1-RFP accumulation.

bioRxiv preprint doi: https://doi.org/10.1101/2023.02.14.528546; this version posted February 14, 2023. The copyright holder for this preprint (which was not certified by peer review) is the author/funder, who has granted bioRxiv a license to display the preprint in perpetuity. It is made available under aCC-BY 4.0 International license.

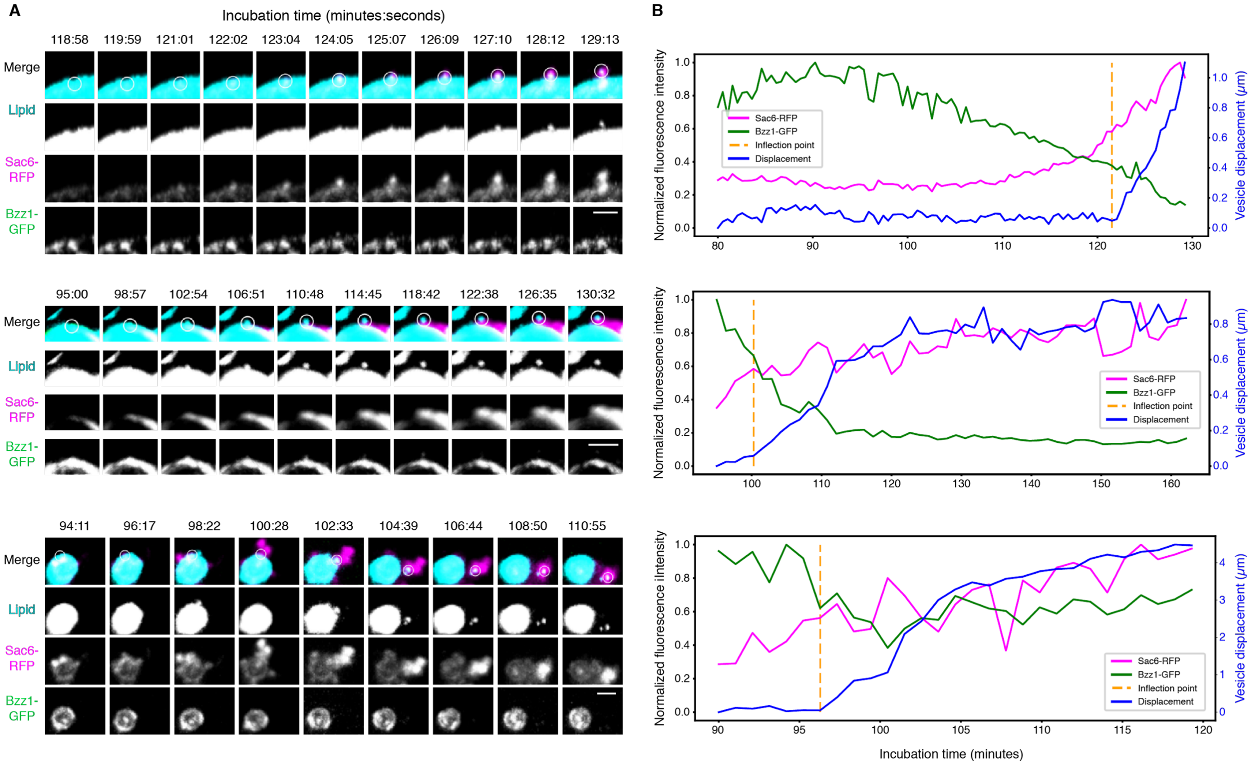

Figure S4. scWASP-coated supported lipid bilayers (with 2% Atto647-DOPE) were incubated in Bzz1-GFP Sac6-RFP cytoplasmic extract for the indicated times. (A) Montages of vesiculation events on 5 µm (upper two panels) and 2 µm (lower panel) beads. A maximum intensity projection of a substack through the center of the supported lipid bilayer is displayed for all channels. The white circle in the merged channel indicates the region used for quantitative analysis. Scale bars, 2 µm. (B) Quantification traces of fluorescence intensities and vesicle displacement from the vesiculation events visualized in (A).

bioRxiv preprint doi: https://doi.org/10.1101/2023.02.14.528546; this version posted February 14, 2023. The copyright holder for this preprint (which was not certified by peer review) is the author/funder, who has granted bioRxiv a license to display the preprint in perpetuity. It is made available under aCC-BY 4.0 International license.

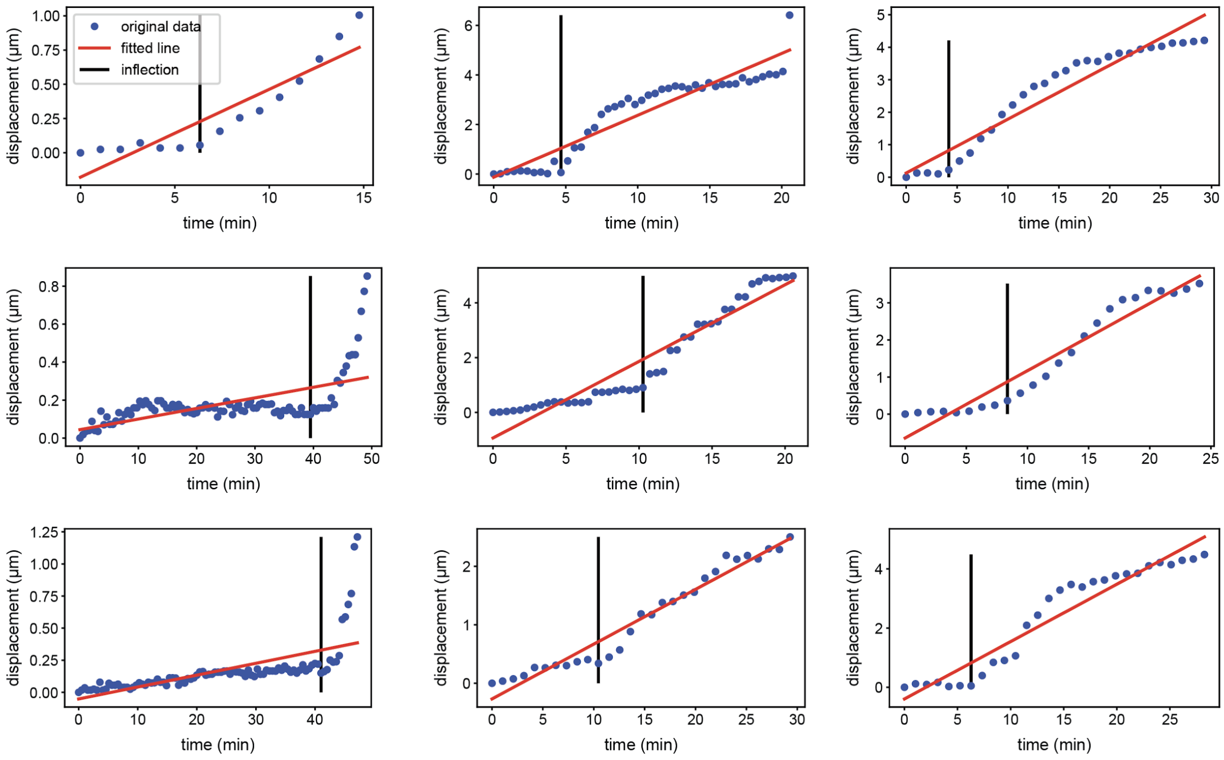

Figure S5. Gallery of randomly selected individual vesiculation event traces used for inflection point calculations. Blue dots represent the vesicle displacement in 3D volume from the origin position at the beginning of the event. A linear regression was fit to each vesiculation event trace. The inflection point was then determined as the time point at which the vesicle displacement was the maximum negative difference from the linear regression, out of all the time points before the point at the maximum positive difference from the linear regression. The constraint for points before the maximum positive difference from the linear regression was necessary to filter out points in displacement traces that plateaued at later time points.

bioRxiv preprint doi: https://doi.org/10.1101/2023.02.14.528546; this version posted February 14, 2023. The copyright holder for this preprint (which was not certified by peer review) is the author/funder, who has granted bioRxiv a license to display the preprint in perpetuity. It is made available under aCC-BY 4.0 International license.

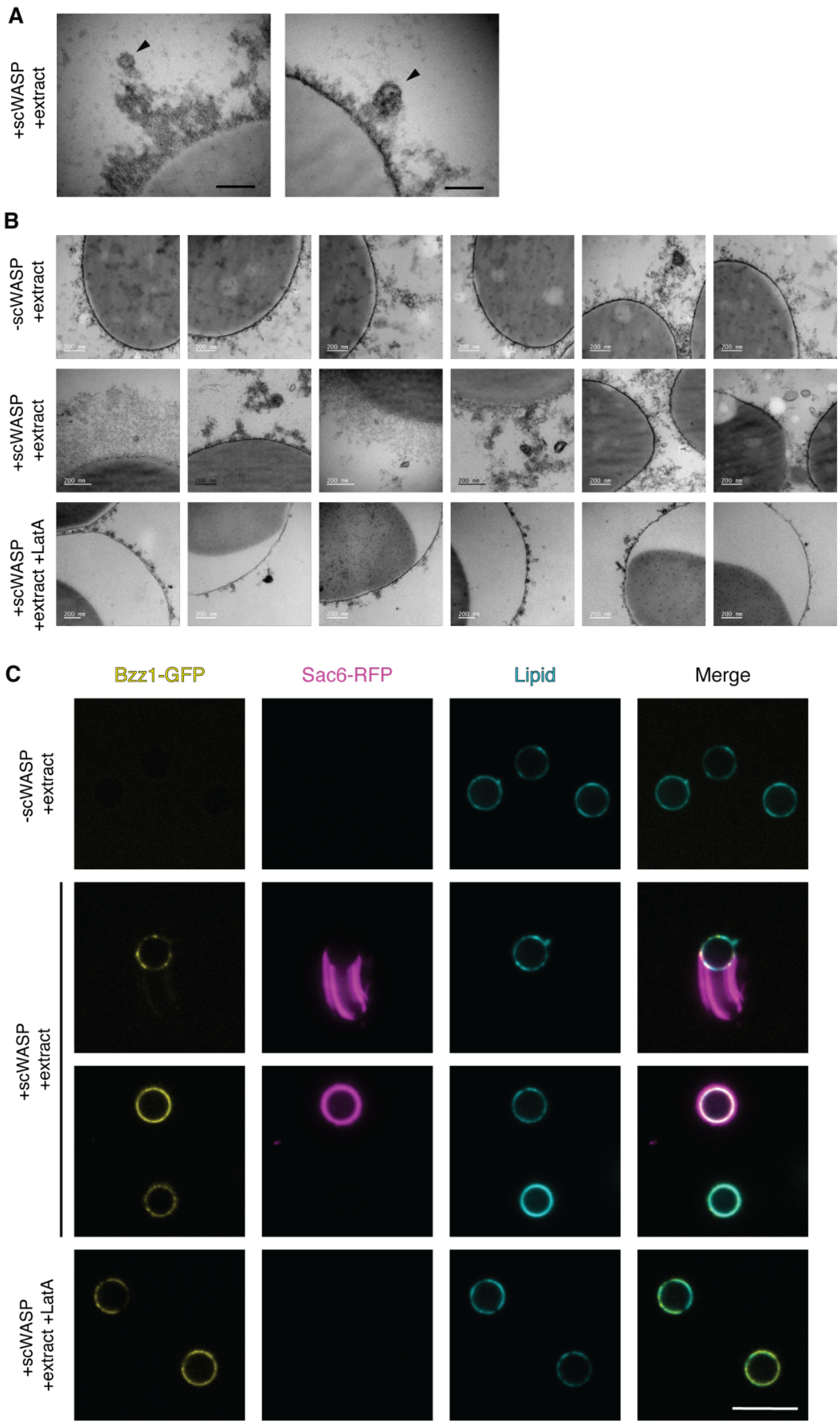

bioRxiv preprint doi: https://doi.org/10.1101/2023.02.14.528546; this version posted February 14, 2023. The copyright holder for this preprint (which was not certified by peer review) is the author/funder, who has granted bioRxiv a license to display the preprint in perpetuity. It is made available under aCC-BY 4.0 International license.

Figure S6. (A) High magnification EM images of vesicles (arrowheads) observed around supported lipid bilayers coated with scWASP and incubated in cytoplasmic extract. Scale bar, 100 nm. (B) Supported lipid bilayers on microbeads were either coated with or not coated with scWASP and incubated in cytoplasmic extract with or without latrunculin A, as indicated. Representative EM images of membrane deformations observed for each condition are presented here. (C) scWASP-coated bilayers were incubated in cytoplasmic extract from a strain expressing Bzz1-GFP and Sac6-RFP. Single confocal slices at the midsection of scWASP-coated bilayers containing lipid dye (2% Atto647-DOPE) are displayed. Scale bar, 10 µm.

bioRxiv preprint doi: https://doi.org/10.1101/2023.02.14.528546; this version posted February 14, 2023. The copyright holder for this preprint (which was not certified by peer review) is the author/funder, who has granted bioRxiv a license to display the preprint in perpetuity. It is made available under aCC-BY 4.0 International license.

Table S1. Strains used in this study.

DDY Strain	Genotype	Source
DDY5783	MATa ade2 leu2 his3 trp1 ura3 lys2::Pgal1-GAL4 pep4::HIS3 bar1::hisG	St-Pierre et al 2009
DDY5784	MATa his3-Δ200, leu2-3, 112, ura3-52, SAC6-RFP::KANmx	This study
DDY5785	MATα his3-Δ200, leu2-3, 112, lys2-801 ura3-52, RVS167-GFP::HIS3 SAC6-RFP::KANmx	This study
DDY5786	MATα his3-Δ200, leu2-3, 112, ura3-52, MYO5-GFP::HIS3 SAC6-RFP::KANmx	This study
DDY5787	MATα his3-Δ200, leu2-3, 112, ura3-52, ARK1-GFP::KANmx SAC6-RFP::KANmx	This study
DDY5788	MATα his3-Δ200, leu2-3, 112, ura3-52, lys2-801, VRP1-GFP::HIS3 SAC6-RFP::KANmx	This study
DDY5789	MATα his3-Δ200, leu2-3, 112, ura3-52, BZZ1-GFP::HYGmx SAC6-RFP::KANmx	This study
DDY5790	MATα his3-Δ200, leu2-3, 112, ura3-52, lys2-801, BBC1-GFP::HIS3 SAC6-RFP::KANmx	This study
DDY4350	MATα his3-Δ200, leu2-3, 112, ura3-52, ABP140-3×GFP::HIS3	Miao et al 2013
DDY5791	MATα his3-Δ200, leu2-3, 112, ura3-52, SLA2-GFP::HIS3 SAC6-RFP::KANmx	This study
DDY2960	MATα his3-Δ200, leu2-3, 112 lys2-801, ura3-52, SAC6-GFP::HIS3	Martin et al 2006
DDY3937	MATα his3-Δ200, leu2-3, 112, ura3-52, BZZ1-GFP::HygMX, RVS167-RFP::HIS3	Kishimoto et al 2011

bioRxiv preprint doi: https://doi.org/10.1101/2023.02.14.528546; this version posted February 14, 2023. The copyright holder for this preprint (which was not certified by peer review) is the author/funder, who has granted bioRxiv a license to display the preprint in perpetuity. It is made available under aCC-BY 4.0 International license.

Table S2. **Manual quantification of the occurrence of actin-associated lipid protrusions on bilayers of differing lipid compositions.** Where indicated, 20% phosphatidylserine (PS) and/or 5% phosphatidylinositol 4,5 bisphosphate (PI(4,5)P_2) were added to bilayers containing 5% DGS-NTA, 2% ATTO 647-DOPE, and phosphatidylcholine (PC) up to 100%.

Lipid composition	n beads	Beads with actin-associated vesiculation	Percent
PC	103	3	0.029
PC + PS	154	11	0.071
PC + PI(4,5)P_2	100	20	0.200
PC + PS + PI(4,5)P_2	115	22	0.191

bioRxiv preprint doi: https://doi.org/10.1101/2023.02.14.528546; this version posted February 14, 2023. The copyright holder for this preprint (which was not certified by peer review) is the author/funder, who has granted bioRxiv a license to display the preprint in perpetuity. It is made available under aCC-BY 4.0 International license.

Table S3. **Quantification of vesicle budding events observed in time lapse imaging.** For each condition listed, the number of fluorescent vesicles formed from the surface of bead SLBs (budding events) was manually counted over the course of 30- to 60-minute 3D time-lapse experiments. The number of budding events per experiment was divided by the number of beads and averaged among replicate experiments to calculate the mean and standard deviation budding events per bead.

Condition	n beads	Mean budding events per bead	Standard deviation
-scWASP -LatA +extract	49	0.0476	0.0825
+scWASP -LatA +extract	97	0.248	0.291
+scWASP +LatA +extract	188	0.0481	0.0638
+scWASP -LatA -extract	16	0	0

bioRxiv preprint doi: https://doi.org/10.1101/2023.02.14.528546; this version posted February 14, 2023. The copyright holder for this preprint (which was not certified by peer review) is the author/funder, who has granted bioRxiv a license to display the preprint in perpetuity. It is made available under aCC-BY 4.0 International license.

Table S4. **Quantification of vesicles observed by EM.** For each condition listed, the number of vesicles and membrane structures at given distances away from the bead surface was manually counted using 2D electron micrographs. Events were categorized as 'adjacent' if within 100 nm of the bead surface or 'nonadjacent' if between 100 nm and 1000 nm from the bead surface. Vesicles found greater than 1000 nm from a particular bead were not counted.

Condition	n beads	Adjacent events (lipid bilayers 10 - 100 nm from bead surface)	Adjacent events per bead	Nonadjacent events (lipid bilayers 100 - 1000 nm from bead surface)	Nonadjacent events per bead
-scWASP -LatA +extract	47	3	0.0638	2	0.0426
+scWASP -LatA +extract	51	11	0.216	32	0.627
+scWASP +LatA +extract	43	36	0.837	7	0.163
+scWASP -LatA -extract	16	0	0	0	0

bioRxiv preprint doi: https://doi.org/10.1101/2023.02.14.528546; this version posted February 14, 2023. The copyright holder for this preprint (which was not certified by peer review) is the author/funder, who has granted bioRxiv a license to display the preprint in perpetuity. It is made available under aCC-BY 4.0 International license.

Movie S1. 3D rendering of actin tail assembled on scWASP-coated bilayer. Related to Figure 1D. scWASP-coated supported lipid bilayers were incubated in cytoplasmic extract expressing Sac6-RFP. A 3D rendering of a confocal stack is displayed. Blue outline indicates bead location. Scale bars, 5 µm.

Movie S2. Time-lapse and 3D rendering of sequential recruitment of endocytic proteins to scWASP-coated supported bilayers. Related to Figure 2D. scWASP-coated supported lipid bilayers were incubated in Bzz1-GFP (green) Sac6-RFP (magenta) cytoplasmic extracts. A 3D rendering of a substack of the bead is displayed. Time after addition of extract to bilayers is displayed. Frames were generated every 57 s and are played back at 6.7 fps. Scale bars, 5 µm.

Movie S3. 3D rendering of a vesicle embedded in an actin tail. Related to Figure S3. scWASP-coated supported lipid bilayers were incubated in cytoplasmic extracts from cells expressing Bzz1-GFP (not displayed) and Sac6-RFP (magenta). Bilayers include 2% Atto647-DOPE (cyan) to allow visualization of lipid. A 3D rendering of a static confocal stack of the bead is displayed. The white circle indicates location of a diffraction-limited vesicle embedded in an actin tail. Scale bars, 5 µm.

Movie S4. 3D rendering of an actin-associated vesicle in the vicinity of a supported lipid bilayer. Related to Figure S3. scWASP-coated supported lipid bilayers were incubated in cytoplasmic extracts from cells expressing Bzz1-GFP (not displayed) and Sac6-RFP (magenta). Bilayers include 2% Atto647-DOPE (cyan) to allow visualization of lipid. A 3D rendering of a static confocal stack of the bead is displayed. The white circle indicates the location of diffraction-limited, actin-associated vesicles. Scale bars, 5 µm.

bioRxiv preprint doi: https://doi.org/10.1101/2023.02.14.528546; this version posted February 14, 2023. The copyright holder for this preprint (which was not certified by peer review) is the author/funder, who has granted bioRxiv a license to display the preprint in perpetuity. It is made available under aCC-BY 4.0 International license.

Movie S5. 3D rendering of reconstitution of actin-mediated vesicle budding. Related to Figure 3A. scWASP-coated supported lipid bilayers were incubated in Bzz1-GFP (green) Sac6-RFP (magenta) cytoplasmic extracts. Bilayers include 2% Atto647-DOPE (cyan) to allow visualization of lipid. A 3D rendering of a substack of the bead is displayed. The white circle indicates the region of vesicle budding. A white line traces the path of the vesicle. Time after addition of extract to bilayers is displayed. Frames were generated every 57 s and are played back at 6.7 fps. Scale bars, 5 µm.

Movie S6. 3D rendering of actin-mediated vesicle budding from supported lipid bilayer on 5 µm bead. Related to Figure S4, upper panel. scWASP-coated supported lipid bilayers were incubated in Bzz1-GFP (green) Sac6-RFP (magenta) cytoplasmic extracts. Bilayers include 2% Atto647-DOPE (cyan) to allow visualization of lipid. A 3D rendering of a substack of the bead is displayed. The white circle indicates the region of vesicle budding. A white line traces the path of the vesicle. Time after addition of extract to bilayers is displayed. Frames were generated every 30.8 s and are played back at 6.7 fps. Scale bars, 5 µm.

Movie S7. 3D rendering of actin-mediated vesicle budding from supported lipid bilayer on 5 µm bead. Related to Figure S4, middle panel. scWASP-coated supported lipid bilayers were incubated in Bzz1-GFP (green) Sac6-RFP (magenta) cytoplasmic extracts. Bilayers include 2% Atto647-DOPE (cyan) to allow visualization of lipid. A 3D rendering of a substack of the bead is displayed. The white circle indicates the region of vesicle budding. A white line traces the path of the vesicle. Time after addition of extract to bilayers is displayed. Frames were generated every 79 s and are played back at 6.7 fps. Scale bars, 5 µm.

bioRxiv preprint doi: https://doi.org/10.1101/2023.02.14.528546; this version posted February 14, 2023. The copyright holder for this preprint (which was not certified by peer review) is the author/funder, who has granted bioRxiv a license to display the preprint in perpetuity. It is made available under aCC-BY 4.0 International license.

Movie S8. 3D rendering of actin-mediated vesicle budding from supported lipid bilayer on 2 µm bead. Related to Figure S4, lower panel. scWASP-coated supported lipid bilayers were incubated in Bzz1-GFP (green) Sac6-RFP (magenta) cytoplasmic extracts. Bilayers include 2% Atto647-DOPE (cyan) to allow visualization of lipid. A 3D rendering of a substack of the bead is displayed. The white circle indicates the region of vesicle budding. A white line traces the path of the vesicle. Time after addition of extract to bilayers is displayed. Frames were generated every 62.8 s and are played back at 6.7 fps. Scale bars, 5 µm.
